# Restoration of Turbid Underwater Images of Cobalt Crusts Using Combined Homomorphic Filtering and a Polarization Imaging System

**DOI:** 10.3390/s25041088

**Published:** 2025-02-11

**Authors:** Enzu Peng, Chengyi Liu, Haiming Zhao

**Affiliations:** 1 The College of Mechanical and Electrical Engineering, Central South University, Changsha 410083, China; 8204222401@csu.edu.cn (E.P.);; 2 State Key Laboratory of High Performance Complex Manufacturing, Central South University, Changsha 410083, China

**Keywords:** homomorphic filtering, polarization imaging, backscattered light, imaging through turbid media, cobalt crust

## Abstract

Marine cobalt-rich crusts, extensively used in industries such as aerospace, automotive, and electronics, are crucial mineral resources located on the ocean floor. To effectively exploit these valuable resources, underwater imaging is essential for real-time detection and distribution mapping in mining areas. However, the presence of suspended particles in the seabed mining environment severely degrades image quality due to light scattering and absorption, hindering the effective identification of the target objects. Traditional image processing techniques—including spatial and frequency domain methods—are ineffective in addressing the interference caused by suspended particles and offer only limited enhancement effects. This paper proposes a novel underwater image restoration method that combines polarization imaging and homomorphic filtering. By exploiting the differences in polarization characteristics between suspended particles and target objects, polarization imaging is used to separate backscattered light from the target signal, enhancing the clarity of the cobalt crust images. Homomorphic filtering is then applied to improve the intensity distribution and contrast of the orthogonal polarization images. To optimize the parameters, a genetic algorithm is used with image quality evaluation indices as the fitness function. The proposed method was compared with traditional image processing techniques and classical polarization imaging methods. Experimental results demonstrate that the proposed approach more effectively suppresses backscattered light, enhancing the clarity of target object features. With significant improvements in image quality confirmed by several no-reference quality metrics, the method shows promise as a solution for high-quality underwater imaging in turbid environments, particularly for deep-sea mining of cobalt-rich crusts.

## 1. Introduction

Cobalt is an important mineral resource widely used in aerospace, aviation, automotive, chemical, ceramics, and other industries. Marine cobalt crust resources are richer than those on land, mainly existing attached to bedrock, but the surface coverage is only about 50% [1]. With continuous surveys since the end of the last century, research on seafloor cobalt crusts has shifted from mining area detection to exploration and extraction [2]. Real-time identification of cobalt crusts is a crucial prerequisite for mining; therefore, accurately detecting and identifying the distribution of deep-sea cobalt crust deposits is of great significance.

Currently, underwater target detection mainly employs two methods: sonar detection and optical imaging. Sonar detection has the advantages of a wide detection range and strong anti-interference capability, but, due to limitations in the number of beams and recognition accuracy, the efficiency of substrate identification within the area is not high. Optical imaging methods can obtain richer and more intuitive information, enhancing recognition efficiency and accuracy. However, during actual mining operations, sediment stirred up by mining equipment can significantly interfere with imaging effects, causing images to become blurred and “foggy”, leading to the loss of target details. To achieve precise excavation and minimize the image degradation effects caused by suspended particles, further research on imaging technology in turbid underwater environments is necessary.

Underwater image processing technologies are mainly divided into image enhancement techniques based on pixel transformation and image restoration methods based on degradation models. Common methods include histogram-based processing, Retinex theory-based methods, image fusion, and deep learning-based underwater image enhancement [3]. These methods process images by directly manipulating pixels or by inversely solving degradation models, with their effectiveness directly dependent on the original image and the constructed prior model. However, due to the lack of information about actual interference sources and the inability to avoid inherent interference from suspended particles present in the scene, the fundamental problem of reduced image clarity remains unsolved, limiting the ability to restore target details.

In contrast, polarization imaging is based on physical imaging models. By capturing images of the same scene under different polarization states and exploiting the differences in polarization characteristics between scattered light and target signal light, it calculates the degree of polarization of backscattered light and the transmission coefficient. This enables the separation of scattered light from the target signal light, thereby achieving clearer imaging. In 2003 and 2005, Schechner et al. proposed a polarization imaging model and a passive underwater polarization imaging model, respectively [4,5], marking the first introduction of underwater imaging de-scattering models. However, in practical applications, due to the high attenuation coefficient in underwater environments and the weakness of natural illumination, imaging requirements are not met, researchers began adopting active illumination in polarization imaging systems. Treibitz et al. [6] proposed an active polarization de-scattering model in turbid water. One of the assumptions of this method is that the degree of polarization of background scattered light is constant. Under active illumination, the light field is non-uniform, leading to an uneven spatial distribution of the degree of polarization of background scattered light in the scene. This makes it difficult to fully suppress backscattered light, and issues of image detail degradation and uneven illumination still exist.

To more accurately calculate the spatial distribution of the degree of polarization of scattered light and improve imaging quality, researchers have studied this from different perspectives. Liu et al. [7] proposed an underwater image restoration method based on Stokes decomposition, but this method requires orthogonal polarized illumination, increasing the requirements for experimental equipment. Li et al. [8] obtained the degree of polarization of experimental objects and background scattered light using optimization algorithms but ignored the global differences in the degree of polarization within the image. Hu et al. [9] removed the light from the target object area of the polarized image and used information from the background area to perform polynomial fitting on the light intensity and degree of polarization in the target object area, estimating a more accurate global degree of polarization value. However, the experiments in this study were conducted in a water tank constructed with acrylic plates, the camera’s shooting range was small, and the target objects were simple, with low grayscale levels used. This differs significantly from wide water environments, limiting the applicability of the method. Wang et al. [10] suppressed the impact of active illumination non-uniformity through frequency-domain homogenization and polarization-weighted fusion. However, the local DoLP-based weight calculation inadequately corrected the global spatial distribution differences in the polarization degree of scattered light, which may result in residual gradient effects under complex light fields. Shen et al. [11] mitigated scattering and illumination non-uniformity through iterative polarization optimization. However, the globally uniform scattering correction parameters failed to adapt to local polarization attenuation variations in complex substrates, which may lead to reduced stability in detail reconstruction for highly heterogeneous seabed regions. Li et al. [12] mitigated underwater illumination non-uniformity using polarizing lenses and averaging filters. But, the fixed parameter set for scattering light correction failed to adapt to spatial variations in complex seabed environments, resulting in unstable scattering suppression performance within high-turbidity zones.

Homomorphic filtering (HF) combines frequency filtering and spatial gray-level transformation. Based on the illumination–reflectance model of images as the foundation for frequency domain processing, setting appropriate parameters can compress the global brightness range, meeting image restoration requirements in complex scenes. Therefore, to mitigate the effects of uneven active illumination and address the uneven distribution of the global degree of polarization in scattering in images without significantly increasing system complexity, this paper incorporates homomorphic filtering into the classical polarization restoration method. By applying homomorphic filtering to the original orthogonally polarized images, the contrast of the images is enhanced, and the light intensity is balanced. While maintaining the polarization relationship is unchanged, the corresponding polarization images are obtained, reducing spatial differences in the degree of polarization and suppressing backscattered light. After optimizing various parameters using a genetic algorithm, the polarization restoration algorithm is employed to ultimately achieve clear imaging in turbid water environments.

## 2. Fundamental Theory

### 2.1. Underwater Imaging Model

The Jaffe–McGlamery model [13] is a classic and commonly used underwater imaging model. This model indicates that the total light intensity received by an underwater imaging system is composed of a linear superposition of direct components, forward scattering components, and backscattering components. In turbid water imaging environments, the primary factor leading to image quality degradation is the interference of backscattered light. Neglecting the forward scattering component, the total light intensity $I\left(x,y\right)$ can be expressed as the sum of the target reflected light D(x,y) and the backscattered light B(x,y):

(1) I(x,y)=D(x,y)+B(x,y)

Among them, the target reflected light $D\left(x,y\right)$ is the original reflected light L(x,y) (the restored image) that enters the camera after absorption and scattering by particles, represented as the product of the original reflected light L(x,y) and the transmittance t(x,y).

The target reflected light $D\left(x,y\right)$ is inversely proportional to the transmittance of the backscattered light B(x,y). Therefore, the backscattered light is expressed as the product of the backscattered light at infinity $A_{\infty }$ (corresponding to the background region in the image) and the transmittance function $\left[1-t(x,y)\right]$:

(2) D(x,y)=L(x,y)t(x,y)

(3) $$B(x,y)=A_{\infty }\left[1-t(x,y)\right]$$

By substituting Equations (2) and (3) into Equation (1) and simplifying, formulas for calculating the transmission rate $t\left(x,y\right)$ and the restored image $L\left(x,y\right)$ are derived.

(4) $$t(x,y)=1-\frac{B(x,y)}{A_{\infty }}$$

(5) $$L(x,y)=\frac{I(x,y)-B(x,y)}{t(x,y)}$$

### 2.2. Orthogonally Polarized Images

After passing through a fixed linear polarizer (polarizer), the active light source becomes linearly polarized light. By rotating the linear polarizer (analyzer) in front of the camera lens, orthogonally polarized images of the same scene can be captured. When the analyzer is aligned parallel to the polarizer, the maximum intensity image $I^{∥}(x,y)$ is obtained; when it is perpendicular, the minimum intensity image $I^{⊥}(x,y)$ is obtained. The total intensity I(x,y) is the sum of these two images, combined with Equation (1):

(6) $$I(x,y)=I^{∥}(x,y)+I^{⊥}(x,y)=\left[D^{∥}(x,y)+B^{∥}(x,y)\right]+\left[D^{⊥}(x,y)+B^{⊥}(x,y)\right]$$

Cobalt crusts exhibit low polarization characteristics due to their irregular surfaces and porous medium properties. Assuming that the polarization direction of the target reflected light D(x,y) is completely random (unpolarized), we have this equation:

(7) $$D^{∥}\left(x,y\right)=D^{⊥}\left(x,y\right)$$

Under this assumption, the target reflected light components D(x,y) in both the maximum and minimum intensity images are equal. Combining Equations (6) and (7), this can be expressed:

(8) $$I^{∥}(x,y)=\frac{D\left(x,y\right)}{2}+B^{∥}\left(x,y\right)$$

(9) $$I^{⊥}(x,y)=\frac{D(x,y)}{2}+B^{⊥}(x,y)$$

Subtracting Equation (9) from Equation (8) shows that the difference image between the orthogonally polarized images ΔI(x,y) equals the difference in backscattered light ΔB(x,y):

(10) $$\Delta I(x,y)=I^{∥}(x,y)-I^{⊥}(x,y)=B^{∥}(x,y)-B^{⊥}(x,y)=\Delta B(x,y)$$

Due to water interference and the uneven illumination caused by active lighting, the degree of polarization of scattered light $P_{b}(x,y)$ varies across the image. Using only the original orthogonally polarized images $I^{∥}(x,y)$ and $I^{⊥}(x,y)$, traditional polarization restoration methods cannot effectively separate the backscattered light from the target reflected light, making it difficult to achieve satisfactory imaging results.

### 2.3. Homomorphic Filtering Preprocessing

Homomorphic filtering is an image processing method that combines frequency filtering with spatial domain grayscale transformations. Using the illumination–reflectance model of the image as the foundation for frequency domain processing, it can compress the brightness range and enhance contrast. This approach can overcome the shortcomings of traditional polarization restoration methods to a certain extent without complicating the model. The homomorphic filtering applied to an image can be expressed:

(11) $$I_{hf}(x,y)=\mu^{-1}\left\{\mu \left\{I(x,y)\right\}*\left[(\gamma_{H}-\gamma_{L})H(u,v)+\gamma_{L}\right]\right\}$$

(12) $$H(u,v)=1-\exp\left[-c(D^{2}(u,v)/D_{0}^{2})\right]$$

where $\gamma_{H}$ and $\gamma_{L}$ are the high-frequency and low-frequency gains, respectively; c is the sharpening coefficient; and $D_{0}$ is the cutoff frequency.

A pair of orthogonally polarized images has a fixed polarization relationship, defined by the global degree of polarization P(x,y):

(13) $$P(x,y)=\frac{I^{∥}(x,y)-I^{⊥}(x,y)}{I^{∥}(x,y)+I^{⊥}(x,y)}$$

To maintain the inherent polarization relationship of the orthogonally polarized images after filtering, homomorphic filtering is applied only to the maximum intensity image $I^{∥}(x,y)$ to obtain $I_{hf}^{∥}(x,y)$. The corresponding minimum intensity image $I_{hf}^{⊥}(x,y)$ is then derived using the degree of polarization, ensuring that the degree of polarization of each pair of orthogonally polarized images P(x,y) remains unchanged before and after filtering [14,15]:

(14) $$P(x,y)=\frac{I^{∥}(x,y)-I^{⊥}(x,y)}{I^{∥}(x,y)+I^{⊥}(x,y)}=\frac{I_{hf}^{∥}(x,y)-I_{hf}^{⊥}(x,y)}{I_{hf}^{∥}(x,y)+I_{hf}^{⊥}(x,y)}$$

(15) $$I_{hf}^{⊥}(x,y)=\frac{1-P(x,y)}{1+P(x,y)}I_{hf}^{∥}(x,y)$$

### 2.4. Polarization Restoration

The degree of polarization of the backscattered light in the background region $P_{b}(x,y)$ can be expressed as:

(16) $$P_{b}(x,y)=\frac{B_{hf}^{∥}(x,y)-B_{hf}^{⊥}(x,y)}{B_{hf}^{∥}(x,y)+B_{hf}^{⊥}(x,y)}=\frac{\Delta B_{hf}(x,y)}{B_{hf}(x,y)}=\frac{\Delta I_{hf}(x,y)}{\Delta B_{hf}(x,y)}$$

where $B_{hf}^{∥}(x,y)$ and $B_{hf}^{⊥}(x,y)$ are the horizontally and vertically polarized components of the homomorphic filtered backscattered light, respectively.

The degree of polarization of the backscattered light $P_{b}(x,y)$ is taken from the average value of the background region of the image.

(17) $$P_{b}=average\left[\frac{B_{hf}^{∥}(x,y)+B_{hf}^{⊥}(x,y)}{B_{hf}^{∥}(x,y)-B_{hf}^{⊥}(x,y)}\right]$$

Since $P_{b}$ varies across the background region, it is multiplied by a correction coefficient ε [4] to reduce errors. The backscattered light $B_{hf}(x,y)$ is then calculated:

(18) $$B_{hf}(x,y)=\frac{I_{hf}^{∥}(x,y)-I_{hf}^{⊥}(x,y)}{\varepsilon P_{b}}$$

The backscattered light at infinity $A_{\infty }$ is obtained by averaging the backscattered light in the background region:

(19) $$A_{\infty }=average\left[B_{hf}^{∥}(x,y)+B_{hf}^{⊥}(x,y)\right]$$

Finally, the transmittance t(x,y) and the restored image L(x,y) are calculated:

(20) $$t(x,y)=1-\frac{I_{hf}^{∥}(x,y)-I_{hf}^{⊥}(x,y)}{\varepsilon P_{b}A_{\infty }}$$

(21) $$L(x,y)=\frac{I_{hf}^{∥}(x,y)+I_{hf}^{⊥}(x,y)-\frac{I_{hf}^{∥}(x,y)-I_{hf}^{⊥}(x,y)}{\varepsilon P_{b}}}{t(x,y)}$$

### 2.5. Image Quality Assessment and Optimization

To objectively evaluate the restoration effect, we introduce the image enhancement measure (EME) [16] under no-reference conditions as a quantitative metric for assessing the quality of the restored images:

(22) $$EME=\left|\frac{1}{k_{1}k_{2}}\sum_{k=1}^{k_{1}}\sum_{l=1}^{k_{2}}20\log\left(\frac{i_{\max;k,l}^{\omega }(x,y)}{i_{\min;k,l}^{\omega }(x,y)+q}\right)\right|$$

Here, the image is divided into $k_{1}\times k_{2}$ blocks; $i_{\max;k,l}^{\omega }(x,y)$ and $i_{\min;k,l}^{\omega }(x,y)$ are the maximum and minimum intensities in the ω-th block; and q (set to 0.001) is a infinitesimal value to prevent division by zero. A higher EME value indicates clearer image details and better image quality. Additionally, the EME value serves as the fitness function in the genetic algorithm to optimize the parameters of homomorphic filtering and polarization restoration.

### 2.6. Polarization Image Restoration Procedure

**Figure 1:**
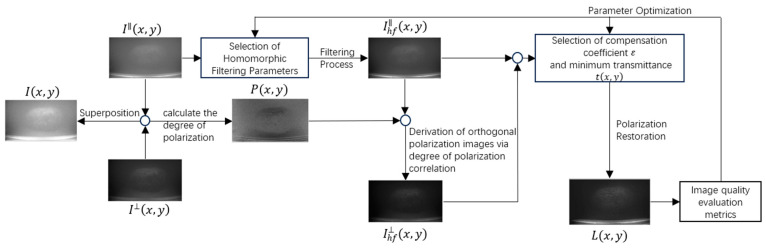
Flowchart of the proposed method in this paper.

The following is a flowchart of the proposed method: see Figure 1.

The method proposed begins by acquiring the original image $I\left(x,y\right)$, along with its horizontal $I^{∥}(x,y)$ and vertical $I^{⊥}(x,y)$ polarization components. The degree of polarization P(x,y) is calculated by superimposing $I^{∥}(x,y)$ and $I^{⊥}(x,y)$. Subsequently, homomorphic filtering is applied to $I^{∥}(x,y)$, resulting in the filtered image $I_{hf}^{∥}(x,y)$. Based on P(x,y) and $I_{hf}^{∥}(x,y)$, the corresponding orthogonal polarization image $I_{hf}^{⊥}(x,y)$ is derived. Polarization restoration is performed by selecting the compensation coefficient ε and minimum transmittance $t\left(x,y\right)$, yielding the final restored image $L\left(x,y\right)$. Finally, image quality evaluation metrics (EME) are employed to assess $L\left(x,y\right)$, the EME assessment also served as fitness function to optimize the parameters of homomorphic filtering and polarization restoration.

## 3. Experimental Design

### 3.1. Water Tank Simulating Mining Environment

**Figure 2:**
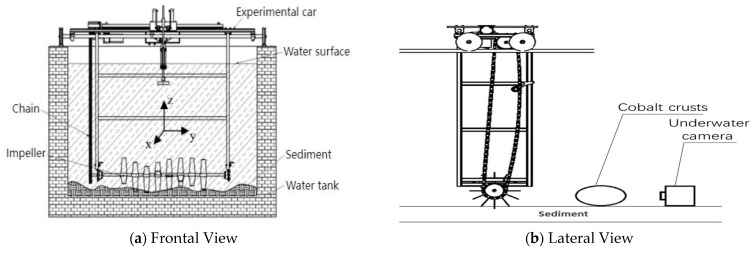
Schematic Diagram of the Experimental Setup. (**a**) is taken from Figure 1a of the article A Volterra series-based method for extracting target echoes in the seafloor mining environment, published in the journal Ultrasonic by Elsevier, and used with permission. Copyright © [18].

Due to the high cost of conducting experiments in a real deep-sea mining environment and to reduce experimental difficulty, a detection experimental system simulating the deep-sea mining environment was designed and established, as shown in Figure 2. The experimental water tank has dimensions of length 5 m, width 3 m, and height 1.8 m. A set of straight-blade impellers positioned 0.3 m above the bottom of the tank was used to simulate the spiral mining head. The impellers were driven to rotate by a reduction motor through chain transmission, stirring the sediments at the bottom of the tank. Fluent 2023R1 software was utilized to simulate the impact of the spiral mining head and multiple impeller models on the underwater flow field, confirming that the experimental model can effectively reflect the real mining environment [17].

### 3.2. Experimental Equipment and Samples

**Figure 3:**
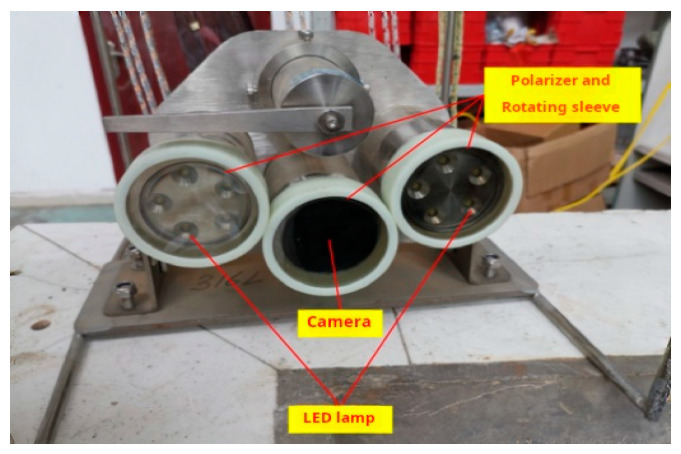
Underwater camera.

**Figure 4:**
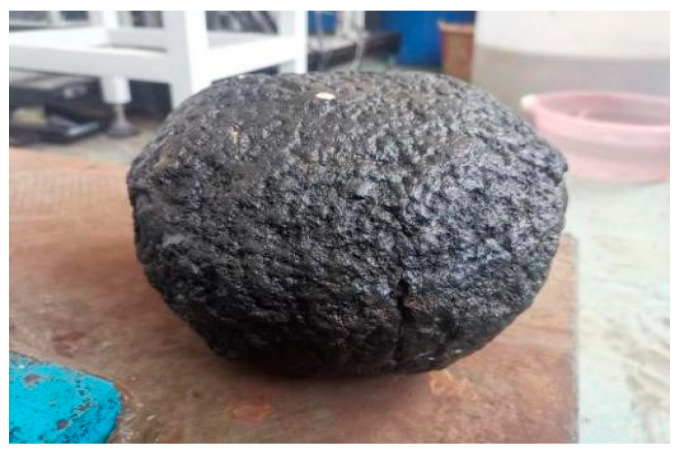
Cobalt crust sample.

The experimental setup utilized the LBF-C50HD3 model three-in-one digital underwater camera manufactured by Robotfish Co., (Qingdao, China), as shown in Figure 3. This system includes an underwater camera (2 megapixels, 1/2.8-inch CMOS sensor, resolution ratio of 1920 × 1080), two LED underwater illumination lights, and a rotating cleaning brush. These components are integrated with linear polarizers to facilitate active polarized light illumination and the acquisition of orthogonally polarized images. The cobalt crust samples used in the experiment, sourced from deep-sea trawl samples as shown in Figure 4, were placed at the bottom of a water tank, 20 cm away from the camera. To simulate sediments, 100 kg of fine river sand with particle sizes of less than 1 mm was pre-deposited at the bottom of the tank.

### 3.3. Image Acquisition

**Figure 5:**
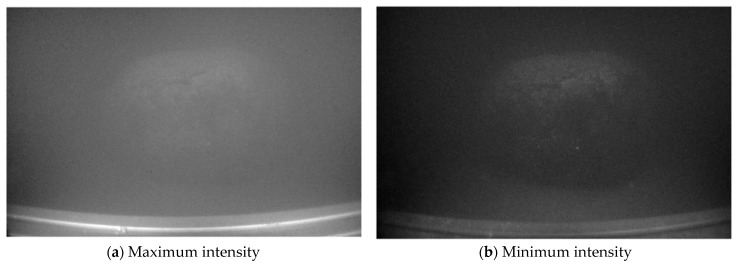

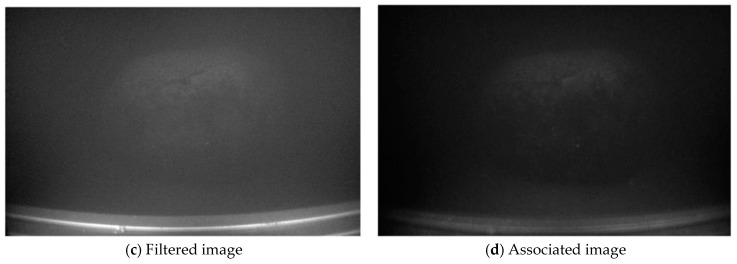
Original orthogonal polarization images and filtered images.

To avoid interference from natural light, the experiment was conducted at night. The impeller rotation speed was set to 30 rpm, and, after stable operation, images of the cobalt crust were captured. By fixing the orientations of the polarizers at the two light sources and rotating the polarizer (analyzer) in front of the lens in Figure 3 to be parallel and perpendicular to polarizers, respectively, the maximum intensity image and the minimum intensity image of the orthogonally polarized images $I^{∥}(x,y)$ and $I^{⊥}(x,y)$ were obtained, as shown in Figure 5a,b. In polarization restoration methods, the key to restoration quality lies in the accurate evaluation of the degree of polarization of backscattered light $P_{b}$ and the backscattered light at infinity $A_{\infty }$. Since the maximum intensity image $I^{∥}(x,y)$ retains most of the backscattered light, applying homomorphic filtering to it can more effectively compress the brightness range of the backscattered light, making subsequent calculations more accurate. Finally, the filtered maximum intensity image $I_{hf}^{∥}(x,y)$ is associated with the global degree of polarization P(x,y) to derive the corresponding minimum intensity image $I_{hf}^{⊥}(x,y)$, as shown in Figure 5c,d. This approach maximizes the suppression of scattered light and highlights the texture details of the target object while maintaining the inherent polarization relationships.

### 3.4. Advantages of the Proposed Method

Compared to existing methods, aside from the differences in principles, the method presented in this paper offers several advantages. Complex experimental setups (such as high-frequency iterative optimization [11]) are not required, and real-time underwater image restoration can be achieved with relatively lower computational resource demands, thus fulfilling the requirements for real-time identification in deep-sea mining operations. Additionally, the experimental system developed in this paper demonstrates a high degree of simulation accuracy, more effectively reflecting image degradation in actual mining environments, thereby enhancing the reliability and adaptability of the method in practical applications.

## 4. Results and Discussion

### 4.1. Parameter Optimization

Homomorphic filtering can simultaneously adjust brightness and improve contrast, but it has many parameters, and the optimal values are related to the specific filtered images. Randomly selecting parameters may result in blurred details and unclear contours; therefore, experimental testing is necessary to determine them. In the polarization restoration process, the selection of the correction coefficient ε in Equation (18) plays a crucial role in the final restoration effect. Additionally, to avoid incorrect estimation of the transmittance t(x,y) in complex environments [19], we imposed a lower bound on the transmittance t(x,y):

(23) $$t(x,y)=\left\{\frac{t(x,y),t(x,y)\geq t_{0}}{t_{0},t(x,y)<t_{0}}\right.$$

Based on the experiments conducted in this manuscript and references [20,21,22,23], the parameter ranges are set as follows: $1<\gamma_{H}<2$, $0<\gamma_{L}<1$, 0<c<10, $0<D_{0}<100$, 1<ε<1.3, and $0<t_{0}<0.2$. Using the EME value as the fitness function, a genetic algorithm is employed to optimize the parameters within these intervals, yielding the optimal values: $\gamma_{H}=1.5$, $\gamma_{L}=0.87$, c=2.48, $D_{0}=49$, ε=1.29 and $t_{0}=0.087$.

### 4.2. Comparison with Schechner’s Method

**Figure 6:**
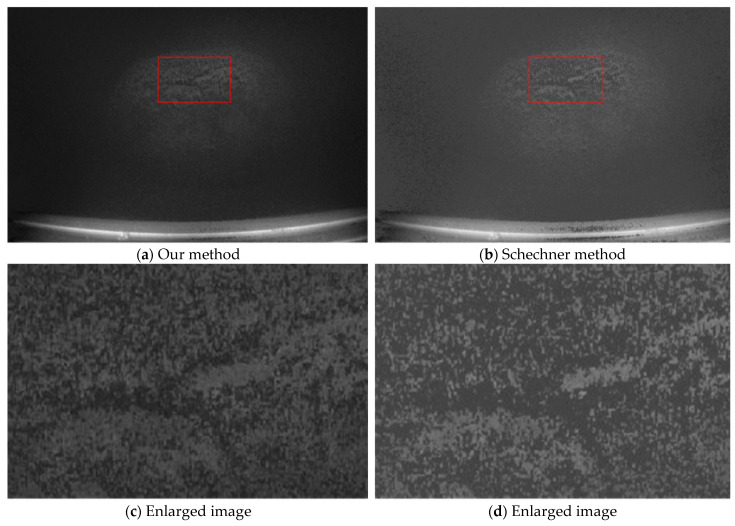
Restoration image comparison.

The restored image obtained using the above parameters is shown in Figure 6a. For comparison, the restored image obtained using the classical Schechner method is also presented in Figure 6b. To facilitate clearer detail comparison and quantitative analysis, the regions within the red boxes in Figure 6a,b are enlarged, as shown in Figure 6c,d.

In Figure 6a, the background region appears pure black due to the suppression of backscattered light, resulting in high contrast with the target object, making the boundary between the target object and the background distinguishable. As seen in Figure 6c, the key texture details of the cobalt-rich crust are richer. The EME values for the images in Figure 6 are calculated as EME(a) = 0.7414, EME(b) = 0.6077, EME(c) = 1.3879, and EME(d) = 1.1125, with EME(a) > EME(b) and EME(c) > EME(d). This analysis indicates that the proposed method suppresses backscattered light more effectively than Schechner’s method, better addressing the issue of uneven backscattered light distribution caused by non-uniform illumination. The overall image quality and target details are both enhanced.

### 4.3. Comparison with Image Enhancement Methods

**Figure 7:**
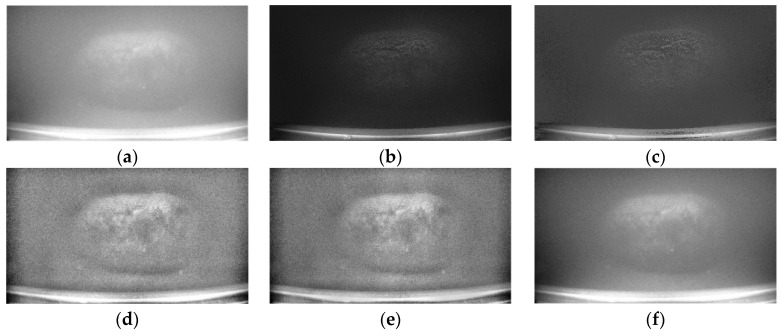
Comparison of Different Methods for Restoring Images. (**a**) Original intensity image method. (**b**) Our method. (**c**) Schechner. (**d**) CLAHE. (**e**) Retinex. (**f**) HF.

To further verify the advantages of the proposed method over other methods, three commonly used image enhancement methods—CLAHE, Retinex, and HF—were employed for comparative study, as shown in Figure 7. There is a significant difference between the images obtained using the polarization restoration method (Figure 7b,c) and those obtained using digital image enhancement methods, manifested in the suppression of scattered light, reduced overall brightness, and significantly enhanced texture details of the target object. Compared with other experiments that use a smaller field of view and increased turbidity using milk [9,23], in the broader water body containing sediment deposits, the background region has uneven scattering and the gray-level variations on the cobalt-rich crust surface are drastic. The CLAHE and Retinex methods not only fail to restore the image but also increase noise in the background region. Although applying HF directly to the original light intensity image improves contrast and image quality to certain extent, the texture details of the cobalt-rich crust are not effectively recovered.

### 4.4. Image Quality Evaluation Metrics

To provide a more comprehensive quantitative analysis of each restored image, in addition to the previously mentioned EME, two other no-reference image quality evaluation metrics were introduced for comprehensive assessment: BRISQUE [24], based on image statistical features, and IC [4], representing the image’s local contrast, as shown in Table 1. It should be noted that higher values of IC and EME indicate better image quality, whereas a lower BRISQUE value signifies better image quality. The EME value is calculated as the average of the ratio function of the maximum and minimum gray-level values within each region of the image, and the IC value depends on the degree of gray-level variation in the image.

However, since the CLAHE and Retinex methods introduce noise, which in turn increases the overall image contrast and results in higher numerical values, they cannot effectively reflect the actual image quality; therefore, they are marked with an asterisk *. Comparing the other methods, it can be seen that the proposed method in this paper achieves the highest numerical values. By analyzing the BRISQUE values of each restored image, it is evident that the restored images using the polarization method demonstrate a substantial improvement over other methods, indicating a substantial enhancement in image quality.

### 4.5. Image Enhancement Under Varying Conditions

**Figure 8:**
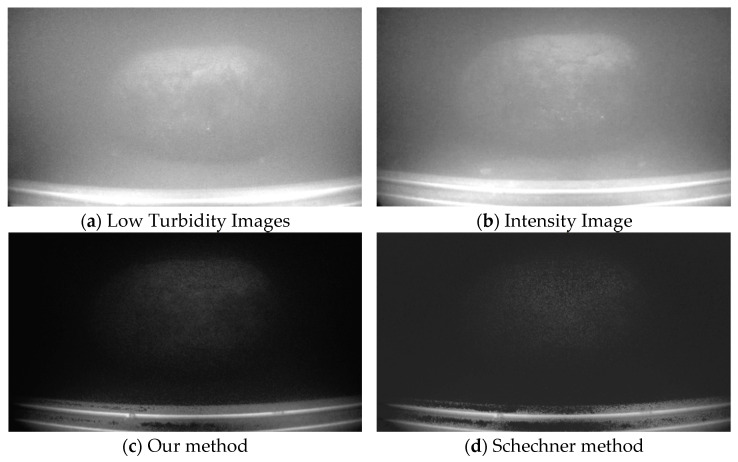
Comparison of restored images with different turbidity levels.

To further validate the robustness and effectiveness of the proposed method in different scenarios, the shooting angle of the cobalt-rich crust was adjusted, and the rotation speed of the impeller was increased to alter the water’s turbidity. After achieving stable operation, polarized images were captured, resulting in high-turbidity images as shown in Figure 8b. In Figure 8c, the scattered light in the background region is suppressed, and the target details have been restored to a certain extent. In Figure 8d, the target boundaries are unclear, and the target exhibits fewer gray levels; compared to Figure 8c, more details are missing. The EME values of each image were calculated as EME(a) = 0.6138, EME(b) = 0.4849, EME(c) = 1.0133, and EME(d) = 0.7949, satisfying the inequality EME(c) > EME(d) > EME(a) > EME(b). These results demonstrate that the proposed method in this paper maintains advantages over classical methods in enhancing image quality.

The sediment particles employed in the experiment exhibit intrinsic consistency with genuine deep-sea suspended particles in optical interference mechanisms. The silicon-oxygen framework structures of quartz/feldspar components in sediments demonstrate less than 8% refractive index discrepancy compared to deep-sea bedrock fragments (pyroxene, plagioclase), with both particle size distributions (sediment D50=18.5 μm, cobalt crust debris D50=22 μm, D50 represents the median diameter of the particles) residing within the scattering-dominated regime [25]. This ensures universal suppression capability against wide-angle scattering noise. For deep-sea-specific metallic oxide fragments (e.g., ferromanganese nodules), the proposed method suppresses strong specular reflections from metallic particles through polarization-based scattering separation mechanism (Equations (5)–(7)). Although constrained by deep-sea in situ experimental conditions, theoretical analysis and characteristic mapping confirm that sediment scattering properties encompass core interference patterns of multiple deep-sea suspended particle types. Combined with the algorithm’s synergistic optimization of polarization disparity and spatial intensity distribution, this substantiates the method’s adaptability to authentic cobalt crust mining environments. Subsequent in situ verification through deep-sea exploration equipment integration should be conducted to enhance performance evaluation frameworks.

### 4.6. Practical Application Potential and Engineering Challenges

The deployment of polarization imaging technology faces challenges in cost and system integration. High-precision polarization cameras and pressure-resistant components significantly increase hardware costs. Additionally, electrical synchronization and mechanical adaptation with existing mining systems (e.g., sonar and robotic arms) are required. The high frame rate needed for polarization image transmission demands a communication bandwidth over 50 Mbps, which necessitates preprocessing through edge computing. Experimental validation indicates scalability for operational ranges of several tens of meters, but further testing is required in complex terrains like fissures and steep slopes. To improve adaptability, lightweight algorithms such as model quantization could enhance edge computing performance.

In terms of mining efficiency, the proposed method improved the image’s EME value by 103%, from 0.6138 to 1.2488, and local contrast by 64%. The BRISQUE score decreased by 31%, indicating a substantial improvement in image quality. Additionally, the improved surface texture and boundary recognition of the cobalt crust allowed for more accurate robotic arm path planning. The integration of sonar data fusion optimized excavation trajectories, while dynamic turbid environments were managed through adaptive parameter updates, maintaining algorithm robustness.

## 5. Conclusions

In this paper, an underwater image restoration method combining digital image processing techniques with polarization imaging is proposed to address the imaging requirements of cobalt-rich crusts in deep-sea mining environments. This method effectively integrates homomorphic filtering with classical polarization restoration and introduces a genetic algorithm to optimize parameter values. Addressing the issue of uneven global polarization degree. Underwater imaging experiments were conducted under different scenarios. The results were compared with other methods using three no-reference image quality evaluation metrics: IC, EME, and BRISQUE. Compared with previous methods, the combination of homomorphic filtering and polarization imaging can effectively suppress scattered light in the background regions of wider water bodies containing sediment deposits, thereby enhancing the contrast of texture details on the surface of the target cobalt-rich crust. Finally, the practical application potential and engineering challenges of this algorithm are analyzed.

Due to experimental limitations, the detection range of the underwater camera was relatively short. Only one cobalt-rich crust sample was placed within the camera’s field of view, and the concentration of suspended particles was relatively uniform. Future research can further explore methods to accurately calculate the global degree of polarization and backscattered light, thereby achieving image restoration in mining environments with non-uniform concentration scenarios.

## Figures and Tables

**Table 1:** Quantitative comparison of recovered images for the images in Figure 7.

	IC	EME	BRISQUE
Intensity image	0.1123	0.6138	35.1301
Our	0.18	1.2488	24.0987
Schechner	0.1478	0.9308	20.6782
HF	0.1360	0.9732	36.5511
CLAHE	0.1966 *	1.4675 *	41.8575
Retinex	0.2153 *	1.7789 *	43.0674

## Data Availability

The data presented in this study are available on request from the corresponding author.
